# Label-Free Two-Photon Spectral Microscopy to Track Black Carbon Fate in Live Copepod

**DOI:** 10.1021/acs.est.5c18449

**Published:** 2026-05-25

**Authors:** Maria L. F. Vicente, Jeanne Blanchet, Mariana M. Veras, Dominique Jamet, Jean-Louis Jamet, Stéphane Mounier, Houssam Hajjoul, Francisco E. G. Guimarães

**Affiliations:** † São Carlos Institute of Physics, University of São Paulo, São Carlos 13566-590, Brazil; ‡ Univ Toulon, Aix Marseille Univ, CNRS, IRD, MIO, Toulon 83041, France; § Laboratório de Patologia Ambiental e Experimental, LIM05 − Hospital das Clinicas, Faculdade de Medicina da Universidade de São Paulo, São Paulo 01246-903, Brasil

**Keywords:** black carbon, dissolved black carbon, zooplankton, *Acartia*, *In vivo* imaging, two-photon microscopy, confocal microscopy

## Abstract

Black carbon (BC), a major product of incomplete combustion, is both a potent climate forcer and a widespread marine pollutant. Yet, its biological interactions remain largely unresolved. Here, we report the first application of two-photon (2P) spectral microscopy to track BC uptake in live zooplankton copepods (*Acartia spp.*). This nonlinear optical technique enables label-free, high-resolution 3D visualization of copepods and uniquely discriminates between their autofluorescence, particulate BC (particulate black carbon (PBC), red-shifted emission ∼650–675 nm), and dissolved/colloidal BC (DBC, broad emission centered near 540 nm) based on intrinsic photophysical fingerprints. By coupling intestinal clearing protocols with spectral mapping, we established, from our knowledge, the first pigment-free intestinal baseline in a live marine zooplankton, eliminating autofluorescence and food-pigment interference. *In vivo* imaging revealed PBC localized along the digestive tract, accompanied by intestinal swelling and a progressive redistribution of spectral weight from PBC to DBC-like emission toward the posterior gut. These findings indicate that soot aggregates undergo partial reorganization under intestinal conditions, generating colloidal fractions not originally abundant in diesel exhaust. Intestinal swelling was quantified using a dimensionless ratio between the midgut width and the body width at the digestive midgut region (d/D), providing a standardized indicator of gut distension associated with feeding and BC ingestion. By resolving the molecular-level organization within soot aggregates under 2P excitation, this methodological advance introduces a new analytical dimension for pollutant-organism studies, extending beyond the particle-scale changes previously reported. 2P spectral microscopy thus emerges as a powerful, label-free probe of aggregation-dependent photophysics *in vivo*, providing new insights into the biological transformation of combustion-derived carbon and its implications for marine carbon cycling.

## Graphical abstract

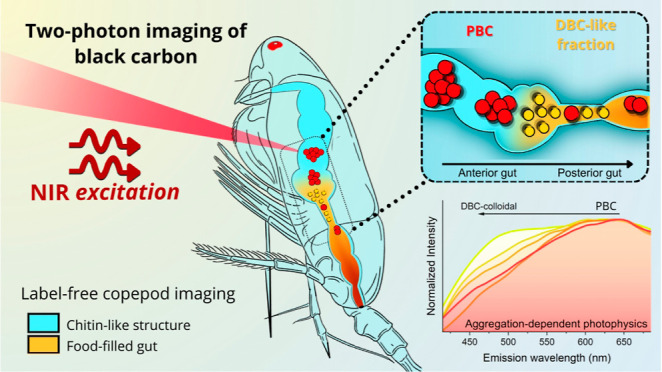

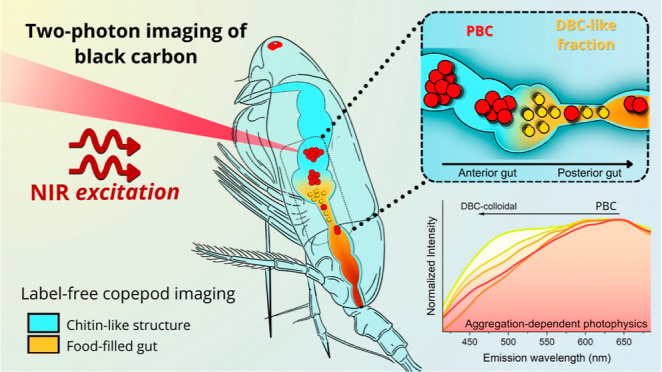

## 1 Introduction

Black carbon (BC) is a ubiquitous anthropogenic pollutant produced by the incomplete combustion of fossil fuels and biomass.^1^ As a potent climate forcer, BC strongly absorbs and scatters sunlight, directly affecting atmospheric heating and radiative balance.^2^ Beyond its climatic influence, BC poses well-documented risks to human health, agriculture, and aquatic ecosystems.^3–7^ Although mainly emitted on land, BC has a short atmospheric lifetime, and a substantial fraction is ultimately transferred to the ocean through atmospheric deposition, rainfall, riverine discharge, or direct release from ship exhaust.^8,9^ In marine environments, BC occurs along a continuum of agglomerate states from particulate soot (particulate black carbon (PBC)) to dissolved or colloidal fractions (DBC), constituting a persistent source of pyrogenic carbon.^10,11^ The DBC pool, composed primarily of polycondensed aromatic molecules, can represent up to ∼20% of dissolved organic matter in lakes and more than 10% in estuarine systems.^12,13^ These highly conjugated structures influence photochemistry and redox processes, inducing oxidative stress and mediating the transformation of organic compounds.^14^ Moreover, recent studies have shown that DBC is bioavailable to marine prokaryotes and that its polycyclic aromatic hydrocarbon (PAH) subunits can bioaccumulate in zooplankton.^15,16^ Together, these findings emphasize the need to elucidate how BC interacts with marine organisms at the microscale. Copepods dominate zooplankton communities, representing nearly 80% of total biomass.^17^ They play a pivotal role in marine food webs, mediating carbon transfer, recycling organic matter, and driving carbon sequestration through fecal pellet sedimentation.^18,19^ Due to their abundance and mixed feeding strategies, copepods are also efficient biological vectors of contaminants, particularly in coastal zones where anthropogenic pollution is highest.^20–22^ However, the nature of copepod–BC interactions remains largely unresolved. This gap limits our ability to assess the pollutant’s effects on marine fauna and to clarify how these organisms contribute to the biotransformation and ultimate fate of BC-derived carbon (e.g., DOM_BC_). Two-photon (2P) microscopy provides a powerful route to bridge this knowledge gap. Unlike conventional single-photon excitation, 2P excitation couples preferentially to extended π-electron systems such as the aromatic domains that dominate BC aggregates. This mechanism enhances spectral selectivity for carbonaceous structures while minimizing photodamage by confining excitation to the focal plane, enabling deep-tissue, nondestructive imaging, due to low scattering and absorption rates in this range. Although 2P microscopy has been applied to tissue analysis and BC detection in biomedical contexts,^23–25^ its potential for environmental imaging, particularly for tracing pollutant–zooplankton interactions, has remained unexplored.^26^ Building on this conceptual framework, we extended 2P spectral microscopy to investigate BC–copepod interactions. This approach established a pigment-free gut baseline, enabling the unequivocal identification of BC signatures *in vivo*. We gathered direct evidence for BC ingestion, redistribution along the digestive tract, and its aggregation-dependent structural reorganization, by spectral evolution observation from particulate to colloidal-like states. Furthermore, the microscopy provided a robust quantitative framework for resolving distinct gut compartments (particularly the midgut B-cell region),^27^ allowing us to use their transverse dimensions as a reliable metric for comparing gut swelling and feeding status. Together, this methodology establishes a new analytical foundation for investigating pollutant–biota interactions and highlights copepods as active biological actors of combustion-derived carbon in marine carbon cycling.

## 2 Methodology

### 2.1 BC Suspension, Fractionation, and Reference Preparation

Diesel exhaust particles were collected directly from a diesel engine using a bimetallic particle retainer designed to trap soot-rich emissions at the source. The sampling vehicle was a 2004 Volkswagen bus equipped with a six-cylinder MWM X-10 engine, operating daily in the Campo Belo district of São Paulo, Brazil (average mileage: 100–150 km·day^–1^). The retainer remained attached to the exhaust for 1 week, and the collected material, rich in elemental carbon, was recovered in September–October 2015.

This direct-source collection prevented atmospheric aging, oxidation, and contamination by urban aerosols, yielding a soot material representative of primary BC emissions. For clarity and consistency, the term BC is used throughout this work to denote the *raw diesel soot material* collected directly from the exhaust, representing a mixed suspension that includes both particulate and dissolved/colloidal fractions. This material, dominated by PBC with minor DBC, served as a standardized reference for reproducible optical and photophysical analyses in both *in vitro* and *in vivo* experiments.

The BC material was provided by the Experimental Air Pollution Laboratory (LIM-05), Department of Pathology, Faculty of Medicine, University of São Paulo, Brazil, following a validated collection protocol.^28^ A stock suspension was prepared by dispersing 1 mg·mL^–1^ of BC in filtered seawater (FSW; 0.7 μm, GF/F) and ultrasonicated for 1 h at 30 °C (40 kHz) to ensure homogeneous dispersion.

The DBC fraction was operationally defined by filtration of the stock suspension through a 0.45 μm hydrophilic cellulose acetate membrane, consistent with the commonly used 0.2–0.7 μm cutoff range to distinguish dissolved/colloidal from particulate BC.^29^ The filtrate was used as the DBC fraction in 2P microscopy experiments, while the unfiltered suspension represented the pristine BC.

To obtain a fraction of PBC (DBC-free), the suspension was centrifuged at medium speed (4000 rpm, 30 min). The resulting pellet was resuspended in FSW, producing a PBC-enriched suspension devoid of soluble or colloidal materials. This fraction was used to evaluate dietary selectivity and ingestion behavior in copepods independent of any DBC contribution.

Together, this multistep preparation yielded three operationally defined carbonaceous materials, BC, PBC, and DBC, each with distinct aggregation states. These fractions formed the experimental basis for subsequent spectral characterization and biological exposure assays.

### 2.2 Two-Photon Microscopy

2P fluorescence imaging was performed using a ZEISS LSM 880 upright laser-scanning microscope equipped with a tunable Ti: sapphire femtosecond laser (690–1100 nm, pulse duration ≈100 fs, repetition rate 80 MHz) and a 32-channel GaAsP cross-calibrated spectral detector array covering 400–700 nm (spectral resolution ≈8–9 nm). In spectral imaging mode, a full emission spectrum was recorded at each pixel, enabling quantitative spectral linear unmixing of endogenous fluorophores and BC signals.

Cross-calibration of the GaAsP detector was performed prior to imaging using the instrument’s spectral equalization routine in ZENsoftware. Detector gain and offset were adjusted to compensate for wavelength-dependent sensitivity, resulting in a linearized spectral response. Periodic supervised calibration checks using spatially homogeneous fluorescence signals were performed to verify a uniform background level and comparable gain across channels.

Unless otherwise stated, an excitation wavelength of 800 nm was used. This wavelength provided efficient 2P excitation of the main intrinsic fluorophores in copepods (chitin-like structures, carotenoids, and chlorophyll-derived pigments) while simultaneously generating detectable emission from BC fractions. Additional exploratory measurements performed between 800 and 1050 nm confirmed that the overall spectral shape of BC emission remained consistent, supporting the robustness of spectral fingerprinting under selected excitation conditions.

Optical characterization of isolated BC and DBC fractions was carried out by spreading aqueous suspensions between hydrophilic coverslips (thickness = 0.15 mm) and imaging with a 40×/1.4 NA oil-immersion objective (pixel dwell time ≈2 μ s). Apparent particle dimensions were estimated from fluorescence intensity line profiles at 611 nm using the full width at half-maximum (fwhm) criterion, acknowledging that the measured widths reflect convolution between particle size and the diffraction-limited point-spread function.

Before ingestion experiments, the aggregation behavior of PBC in filtered seawater (FSW; GF/F, 0.7 μm) was assessed by confocal fluorescence imaging. Suspended PBC formed flocculated aggregates with apparent diameters typically ranging from ∼0.8 to 3 μm, depending on particle concentration and mechanical agitation. In quiescent conditions, these aggregates gradually sedimented, indicating limited colloidal stability in seawater. During prolonged incubations (24–48 h), sedimented aggregates accumulated near the bottom of experimental wells, where they remained accessible to copepod feeding.

For *in vivo* imaging, individual copepods were immobilized in shallow spherical-well chambers, maintaining a thin seawater layer to preserve physiological conditions. Whole-organism imaging was performed with a 10×/0.5 NA objective at a 1024 × 1024 pixel sampling (pixel dwell time ≈ 2.05 μ s). The resulting pixel size represents spatial sampling rather than optical resolution. All the images were performed in a lateral side view orientation of the copepod.

Three-dimensional data sets were acquired by sequential optical sectioning along the *z*-axis (Δ*z* = 4 μ·m; 30–32 sections per specimen), providing sufficient depth to encompass the entire digestive tract. For morphometric analysis of intestinal swelling, the focal plane corresponding to the maximum transverse gut diameter in lateral view was selected to ensure consistent measurement conditions across individuals.

Laser power and acquisition parameters were optimized empirically to avoid detector saturation, photobleaching, optical trapping, or thermally induced artifacts. Under typical imaging conditions (≈0.6 mW average power at the specimen plane, 800 nm excitation), the local fluence delivered per pixel was estimated at ∼0.5 J·cm^–2^ at the focal volume. Considering the dominant scattering contribution, the effective fluence experienced at the particle level was reduced by approximately 2 orders of magnitude (∼10^–2^ J·cm^–2^; see Text S1).

This value remains below reported laser-induced incandescence thresholds for soot sublimation under ultrafast visible excitation (∼4 × 10^–2^ J·cm^–2^ at 1800 K).^1^ Although direct comparison between ns-visible and fs-NIR excitation regimes is not strictly equivalent, the combination of ultrafast pulse duration, subfemtoliter excitation volume, and efficient thermal dissipation in aqueous media strongly limits particle heating. The absence of spectral signatures characteristic of incandescence, as well as the lack of cell bubbling or structural damage during repeated imaging, indicates that detected signals arise from nonlinear photophysical emission processes rather than thermal effects.^30,31^

### 2.3 Sampling, Sorting, and Exposure of Copepods to BC

Zooplankton were collected in April 2024 and September 2025 from the northwestern Mediterranean Sea (Toulon Bay, 43°12′ N, 05°93′ E; Hyères Harbor, 43°07′ N, 06°07′ E) using a 200 μm mesh plankton net for vertical hauls (0–10 m depth). Samples were immediately transferred to insulated containers filled with ambient seawater and transported to the laboratory within 1 h to minimize handling stress. This study used only environmental plankton collected by marine net tows and involved no human samples or vertebrate animals; therefore, ethics approval was not required.

A total of 45 adult *Acartia spp.* ^32^ were randomly sorted using a stereomicroscope from field samples, and sex was not used as a selection criterion. The total of individuals selected comprises 27 *Acartia clausi* and 18 *Acartia tonsa* (≈0.8–1 mm body length). Individuals were distributed into six-well plates containing 10 mL of filtered seawater (FSW; GF/F, 0.7 μm) per well and acclimated for 24 h in the dark at *in situ* temperature to allow gut clearance.

After this clearance period, randomly selected copepods were assigned to exposure conditions consisting of (i) unfiltered seawater (SW), containing natural microalgae and detrital particles, and (ii) FSW enriched with diesel-derived black carbon (BC/PBC, 2.5 mg·L^–1^). The resulting experimental groups were FSW (cleared-gut control; 9 *A. clausi*, 8 *A. tonsa*), SW (food-fed control; 9 *A. clausi*), and BC/PBC exposure (9 *A. clausi*, 8 *A. tonsa*). These control treatments provided baseline spectral and morphometric references for empty versus food-filled intestines. Due to seasonal availability and survival differences after handling, food-fed controls were established only for *A. clausi*.

Although 23 individuals were ultimately analyzed by *in vivo* 2P spectral microscopy, the experimental design prioritized data quality over throughput. Imaging live copepods requires controlled immobilization and acquisition under a thin seawater layer to reduce motion artifacts during spectral scanning. These methodological constraints are inherent to the label-free nonlinear microscopy of motile zooplankton and explain the reduced number of successfully imaged specimens.

### 2.4 Statistical Analyses

Statistical analyses were performed to quantify BC uptake and to evaluate gut reorganization. Differences in intestinal swelling under different experimental conditions were assessed using the ratio between the transverse intestinal diameter (*d*) and the body diameter (*D*) in the midgut B-cell region^27^ (see Supporting Information Figure S3).

Group comparisons were conducted using one-way ANOVA for parametric data sets and Kruskal–Wallis tests followed by Dunn’s multiple comparisons for nonparametric data, across the following conditions: FSW, SW, BC, PBC, cleared, and noncleared guts. Statistical significance was set at *p* < 0.1, consistent with the exploratory nature of the study.

Data on BC/PBC uptake and clearance across experimental groups (FSW, SW, BC, PBC) are presented as mean ± standard error in Table S1 (Supporting Information). Sample sizes (*n*) correspond to the number of individually imaged copepods per group, as specified in the figure legends.

Although only 23 of the 56 prepared copepods were analyzed by 2P spectral microscopy, the experimental setup was optimized to obtain high-quality *in vivo* data. Because imaging live copepods is time-consuming, precise temperature control, and effective immobilization are essential. For this reason, imaging under thin seawater layers was used to minimize motion artifacts during spectral acquisition. These technical constraints are intrinsic to live, label-free 2P microscopy and justify the reduced sample size.

## 3 Results and Discussion

### 3.1 Identification of BC and Endogenous Pigments in *Acartia spp.* Using 2P Microscopy

#### 3.1.1 Reference Spectral Fingerprints of BC Fractions

To establish reference fingerprints for diesel-derived BC fractions, spectral 2P imaging was first applied to seawater suspensions containing the PBC and DBC forms. Emission spectra were obtained from bright spots corresponding to individual particles that naturally deposited on the coverslip while remaining immersed in aqueous droplets (Figure S1A,B).

DBC particles (blue curve in Figure 1A) exhibited a broad emission band centered at ∼550 nm. Notably, this represents the first observation of DBC fluorescence under 2P excitation, demonstrating that the colloidal and particulate components of soot possess measurable nonlinear optical responses whenever electronically coupled domains are present. PBC particles (red curve in Figure 1A) displayed a distinct red-shifted maximum near ∼675 nm, consistent with previous reports of carbon-black emission in biological matrices.^30,33,34^

**Figure 1:**
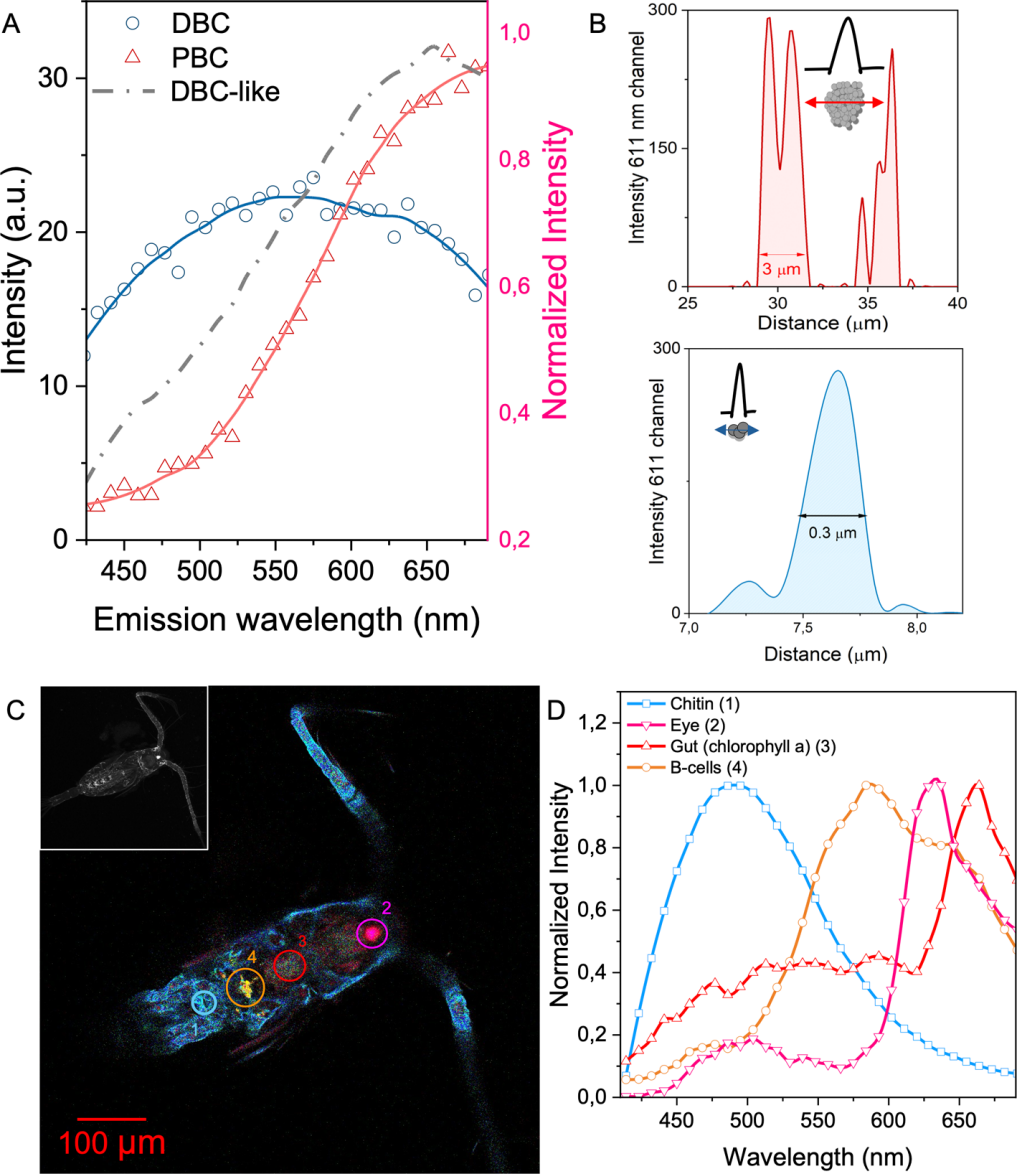
(A) 2P emission spectra of dissolved/colloidal DBC (broad blue line, peak ≈ 550 nm) and particulate PBC (red line, red-shifted to ≈ 675 nm). The simulated DBC-like spectrum (gray dashed line) was obtained by linear superposition of measured PBC and DBC spectra. (B) Intensity profiles across individual PBC (∼0.8–3 μm) and DBC (∼0.3 μm) particles deposited on glass in FSW. (C) Spectral 2P image (ventral view) of a control copepod, with regions of interest (ROIs) marked by colored circles; the inset shows the corresponding widefield image. (D) Representative spectra: chitin (blue, ∼490 nm, ROI 1), B-cells in the midgut (orange, ∼584 nm, ROI 4), carotenoids in the eye spot (magenta, ∼620 nm, ROI 2), and chlorophyll a (red, ∼664 nm, ROI 3). These spectral fingerprints provide reference signatures for distinguishing BC emissions from copepod autofluorescence *in vivo*.

The contrast between the two fractions highlights the photophysical basis for their differentiation. The broad green-centered emission of DBC arises from weakly interacting PAH aggregates, whereas the red-shifted emission of soot-like PBC originates from larger aromatic domains consistent with their multilayered, “onion-like” nanostructure^35,36^ and strong π–π coupling modulated by flexoelectric dipoles.^37^ A key advantage of 2P excitation is its selective sensitivity to aggregated molecular domains. This is clearly evident in soot-like PBC, where highly ordered aromatic stacking enhances the 2P response. In contrast, the less ordered dissolved/colloidal DBC aggregates exhibit weaker intermolecular coupling, yet remain sufficiently electronically correlated to sustain 2P absorption, unlike truly molecularly dissolved PAHs.

To illustrate the potential spectral redistribution arising from simultaneous detection of particulate and colloidal BC domains, a composite reference spectrum was generated by linear superposition of nonnormalized PBC and DBC emission profiles acquired from isolated particles under identical excitation conditions (gray dashed line in Figure 1A). This representation provides a qualitative framework for interpreting *in vivo* spectral evolution but does not constitute a quantitative estimate of relative particle abundance or fluorescence efficiency. The brightness variability among individual particles was observed but did not affect the qualitative spectral shape used for reference fingerprinting. Cross-calibration of the GaAsP spectral detectors ensured that the observed PBC-to-DBC spectral redistribution reflects genuine molecular-level reorganization rather than instrument-specific bias. This methodological rigor reinforces the reliability of 2P spectral microscopy as a physics-based probe for environmental biophotonics.

Particle dimensions were estimated from intensity profiles across individual bright spots at 611 nm (Figure 1B), corresponding to a spectral region common to both PBC and DBC emissions, thereby avoiding wavelength-dependent resolution effects. Fluorescence intensity profiles indicated apparent PBC aggregate dimensions in the range of ∼0.8–3 μm (concentration-dependent; 1.6 ± 0.7 μm reported in Figure 1B), reflecting flocculation of primary soot nanoparticles in seawater, whereas DBC particles averaged ∼0.3 μm. Complementary dynamic-light-scattering (DLS) measurements (Figure S1–C) confirmed that the DBC fraction consisted of particle-like aggregates with hydrodynamic diameters of 200–300 nm, rather than truly molecularly dissolved species.

#### 3.1.2 Endogenous Pigments as a Baseline for Label-Free Copepod Imaging

To discriminate BC emissions from intrinsic copepod autofluorescence, we first characterized the major endogenous pigments of unexposed individuals using label-free 2P spectral imaging (Figure 1C,D). Distinct autofluorescent components were identified at specific ROIs, marked by colored circles on the optical section (ventral view). Chitin, detected in the external body layers (ROI 1, Figure 1C), exhibited a characteristic emission peak at ∼490 nm (blue spectrum, Figure 1D). This pigment is typically abundant in the exoskeleton, digestive tract, and, in variable concentrations, the reproductive tissues of calanoid copepods.^38^ In the eye spot (ROI 2, Figure 1C), carotenoids such as astaxanthin and β-carotene^39,40^ displayed a red-shifted emission maximum near 620 nm under 2P excitation (magenta spectrum, Figure 1D). A displacement of the emission band relative to typical one-photon-excited spectra was observed under two-photon excitation. This behavior is interpreted cautiously as an aggregation-dependent modification of excited-state organization, which is known to influence spectral signatures in conjugated molecular assemblies.^41–43^ Chlorophyll a, detected in the digestive tract (ROI 3, Figure 1C), exhibited its well-known red emission peak at ∼664 nm (red spectrum, Figure 1D).^44^ Additionally, a distinct broad emission centered at ∼584 nm (orange spectrum, Figure 1D) was observed from B-cells in the midgut (ROI 4, Figure 1C). This bright yellowish emission can be assigned to metabolically active materials inside digestive vacuoles that exhibit volume expansion in response to food ingestion. The fluorescence optical response of these B-cells likely varies with enzymatic content and digestive state, as previously described for calanoid copepods.^45^

These well-defined spectral fingerprints, chitin (blue), carotenoids (red), and chlorophyll a (near-infrared), represent the primary autofluorescent components of *Acartia spp.* under 2P excitation. Together, they establish a reliable baseline for distinguishing endogenous emissions from BC-derived fluorescence in subsequent *in vivo* analyses.

#### 3.1.3 Photophysical Mechanisms Underlying BC Emission

The distinct spectral behavior of PBC and DBC under 2P excitation reflects fundamental differences in their aggregation-dependent electronic structure. The red-shifted PBC emission and the green-centered DBC band cannot be attributed to thermal “white-light” generation but instead arise from intrinsic differences in molecular packing and electronic coupling among aromatic domains.^46^ These signatures form the basis for differentiating BC emissions from endogenous copepod autofluorescence.

While 2P excitation is often described macroscopically through nonlinear susceptibilities (χ^2^, χ^3^),^43^ a complementary microscopic view is required to interpret BC behavior. In PBC, tightly packed PAH layers create a dense manifold of electronic states and extended delocalization pathways, increasing the effective 2P absorption cross section. Once excited, strong intermolecular coupling facilitates rapid exciton migration and energy funneling toward low-energy bound states, yielding the characteristic red-shifted emission.^47,48^

In contrast, DBC aggregates display weaker coupling and fewer accessible states, restricting exciton mobility and leading to broader, higher-energy green emission. Thus, 2P excitation acts as a selective probe of molecular organization: ordered PBC aggregates favor red-shifted emission, whereas disordered DBC assemblies produce green fluorescence. This sensitivity, rarely considered in environmental analyses, highlights 2P microscopy not merely as an imaging modality but as a photophysical tool capable of resolving nanoscale order–disorder transitions in combustion-derived carbon.

### 3.2 Localization of Black Carbon within Copepods

#### 3.2.1 *In Vivo* DBC-like Spectral Localization and Gut Baseline Clearance

Figure 2A presents a 3D composite of a live *Acartia* individual (lateral view) incubated for 24 h in FSW enriched with BC suspension (2.5 mg·L^–1^). Image reconstruction used three linearly unmixed spectral channels derived from the reference signatures in the earlier section: Channel 1, chitin emission (light blue); Channel 2, carotenoids and chlorophyll a (orange); and Channel 3, DBC-like emission (red), representing mixed PBC–DBC spectral contributions.

**Figure 2:**
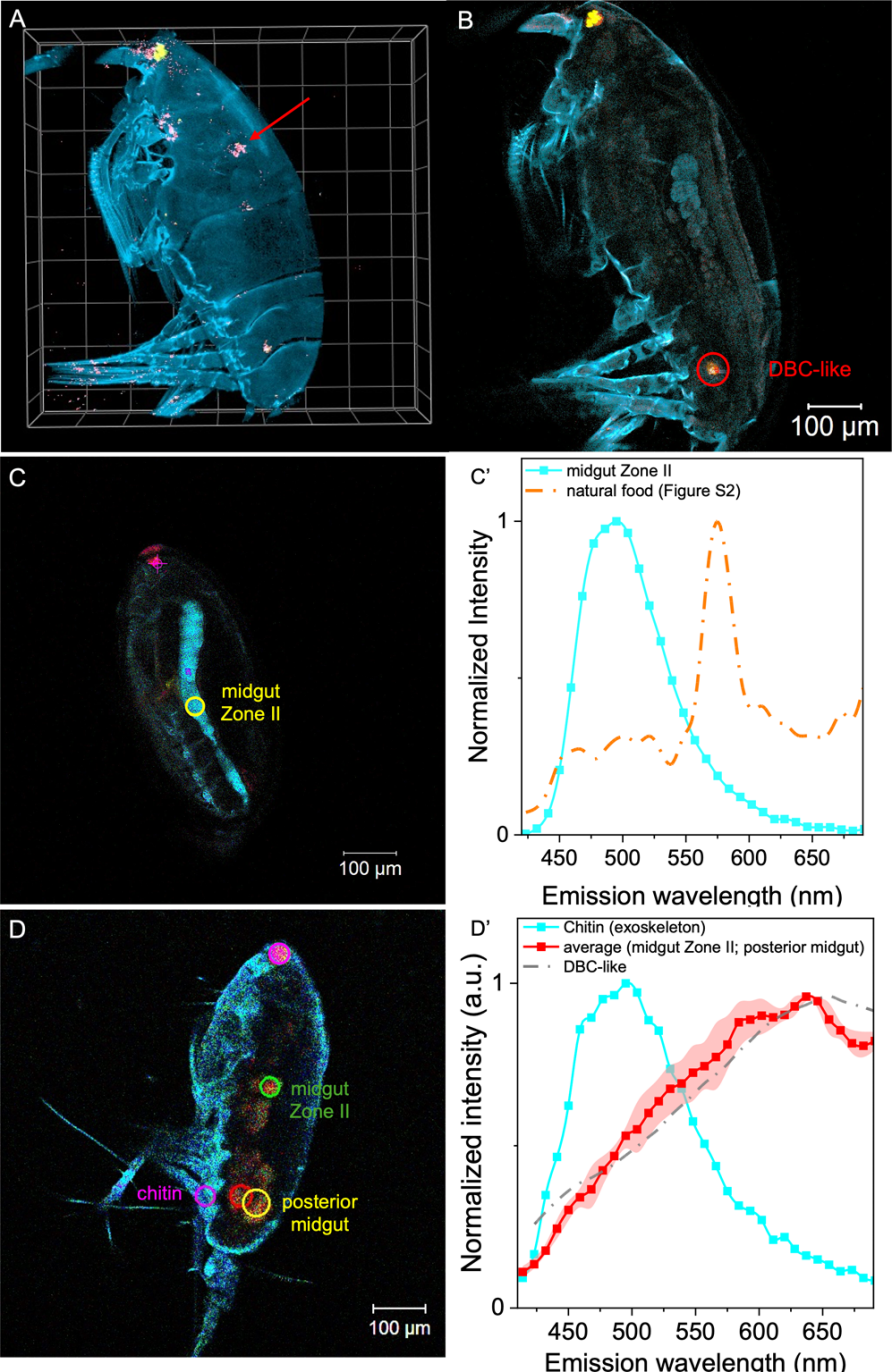
(A) Spectral unmixing image (3D projection, lateral view) of a live *Acartia spp.* incubated for 24 h in BC-enriched seawater (2.5 mg·L^–1^). False colors associated with corresponding spectral signatures: chitin (cyan), carotenoids + chlorophyll a (yellow/orange), and DBC-like domains (red). (B) Optical section from the same data set showing a DBC-like particle localized in the posterior midgut. (C) Side view of a control *Acartia spp.* incubated for 24 h in FSW without BC exposure. (C′) Corresponding spectral profiles for an empty gut (blue line, chitin-like emission at ∼495 nm) and a food-filled gut (orange line, astaxanthin-like emission at ∼577 nm). (D) Lateral spectral image of a copepod exposed to BC (2.5 mg·L^–1^, 24 h) showing intestinal swelling and predominant reddish-yellow emission. (D′) Averaged emission spectrum (red line, ROIs 2–4) obtained from multiple ROIs distributed along the anterior and posterior midgut of a copepod exposed to BC (PBC + DBC), shown together with the corresponding standard-deviation spectral corridor (light-red shading). The reference DBC-like emission profile (gray dash-dot line, from Figure 1A) is superimposed for comparison. The overlap between the *in vivo* spectral variability range and the DBC-like signature supports an aggregation-dependent redistribution of spectral contributions along the digestive tract. The cyan line refers to the chitin reference spectrum (cyan line, ROI 1, exoskeletal surface).

Bright red spots corresponding to DBC-like particles are scattered over the external surface. A distinct internal accumulation was also observed in an optical section of Figure 2A in the posterior gut (Figure 2B), confirming ingestion and internal retention of BC-derived material. External adhesion of marine pollutants to appendages, feeding structures, antennae, and furca has been reported previously and can cause physical interference with locomotion, feeding, reproduction, and mechanoreception.^49–51^

Figure 2C shows a lateral spectral view of a control *Acartia* incubated for 24 h in FSW without BC exposure. The optical section reveals the full intestinal tract, rendered in blue-green by pixel-wise RGB conversion of the spectral data. The intestine appears completely free of food or residual pigments excitable near 400 nm, establishing a clean baseline under 2P excitation. The intestinal diameter ranged from 20 to 40 μm. Spectra from multiple ROI (colored circles, Figure 2C) exhibited identical emission profiles (solid cyan line, Figure 2C′), matching chitin emission with a peak near 495 nm.

For comparison, Figure 2C′ includes the emission spectrum of a food-filled gut (orange line), extracted from a control *Acartia* incubated for 24 h in unfiltered seawater (see Figure S2-A). Spectra acquired from three distinct regions of this full gut, anterior, posterior, and the B-cell area within Midgut Zone II,^27^ were remarkably similar (Figure S2-B). All exhibited a dominant, narrow emission peak at ≈577 nm under 2P excitation, characteristic of astaxanthin-rich dietary material.^39^ This intense yellow band clearly exceeds in weight the weaker chitin-like blue component and the chlorophyll-a red band, giving the full gut its characteristic yellow RGB appearance *in vivo*. Notably, this 577 nm signature constitutes a robust optical marker of active feeding in *Acartia spp.*, independent of gut region, and contrasts sharply with the uniform blue-green chitin-like spectrum observed in the empty gut (Figure 2C–C′).

No additional chromophores were detected, establishing the first pigment-free intestinal baseline for the subsequent comparisons. This clean reference under 2P excitation provides a foundation for assessing spectral and structural signatures associated with BC ingestion.

#### 3.2.2 Ingestion Signatures and Gut Swelling

Figure 2D shows the intestinal tract of a copepod first maintained for 24 h in filtered seawater (FSW) to ensure gut clearance, then incubated for an additional 24 h in BC-enriched seawater (2.5 mg·L^–1^). The side-view RGB spectral image reveals a visibly swollen gut, with lateral diameters ranging from 60 to 80 μ m and a predominant reddish-yellow coloration. Imaging parameters were identical to those used in Figure 2C, allowing direct spectral comparison.

To improve the statistical representation of spectral variability along the digestive tract, averaged emission spectra were calculated from multiple regions of interest (ROIs 2–4; green, red, and yellow circles in Figure 2D) distributed across the anterior and posterior midgut. The resulting mean spectrum, together with the corresponding standard-deviation spectral corridor, reveals a consistent red-shifted emission profile in BC-exposed copepods. Notably, the reference DBC-like spectrum falls within this variability range, indicating that the *in vivo* spectral redistribution is compatible with an increased relative contribution of weakly aggregated or colloidal soot domains rather than the appearance of a distinct new emissive species. The blue-region chitin emission typical of cleared guts was absent.

Together, these findings demonstrate that once ingested, BC particles undergo marked photophysical reorganization within the copepod intestine, yielding emissions consistent with DBC-like domains. From a methodological perspective, these results highlight the necessity of rigorous baselines. The first spectral images of pigment-free copepod intestines (Figure 2C–D) ensured that BC-related emissions were clearly distinguished from endogenous autofluorescence. This “clean-gut” baseline was essential for confirming both ingestion and intestinal responses, such as swelling upon BC exposure.

These controls reinforce the analytical reliability of 2P spectral microscopy and emphasize its potential as a physics-based tool for probing pollutant assimilation and transformation *in vivo*. Finally, the observed spectral redistribution and intestinal distension confirm the ingestion and bioavailability of BC to *Acartia spp.*, which is among the most abundant zooplankton in the oceans. To complement these qualitative findings, we next performed a statistical assessment of midgut swelling to quantify the extent of the ingestion-induced morphological changes.

#### 3.2.3 Quantitative Assessment of Intestinal Swelling

Figure 3 summarizes the intestinal-to-body transverse ratio (d/D), defined as the midgut diameter (d) normalized by the body diameter (D) at the B-cell region. This dimensionless parameter provides a species-independent measure of intestinal swelling and enables comparison between cleared and noncleared guts across treatments. Copepods were grouped as follows: (1) filtered seawater (FSW, food-deprived control); (2) natural seawater (SW, food-rich control); and (3) BC-exposed individuals following 24 h of gut clearance in FSW. For statistical robustness, both *A. clausi* and *A. tonsa* were pooled, as were BC and centrifuged PBC exposures. The BC enrichment concentration used in this study (2.5 mg·L^–1^) exceeds typical environmental levels and was intentionally selected to ensure robust *in vivo* spectral detection within experimentally feasible imaging times. This approach enables reliable volumetric and statistical analysis while establishing a mechanistic proof-of-concept for the fate and photophysical behavior of ingested BC aggregates. Although these concentrations are not intended to reproduce environmental exposure conditions, the observed connectivity-dependent spectral signatures are expected to remain qualitatively valid at lower concentrations, albeit with reduced signal intensity.

**Figure 3:**
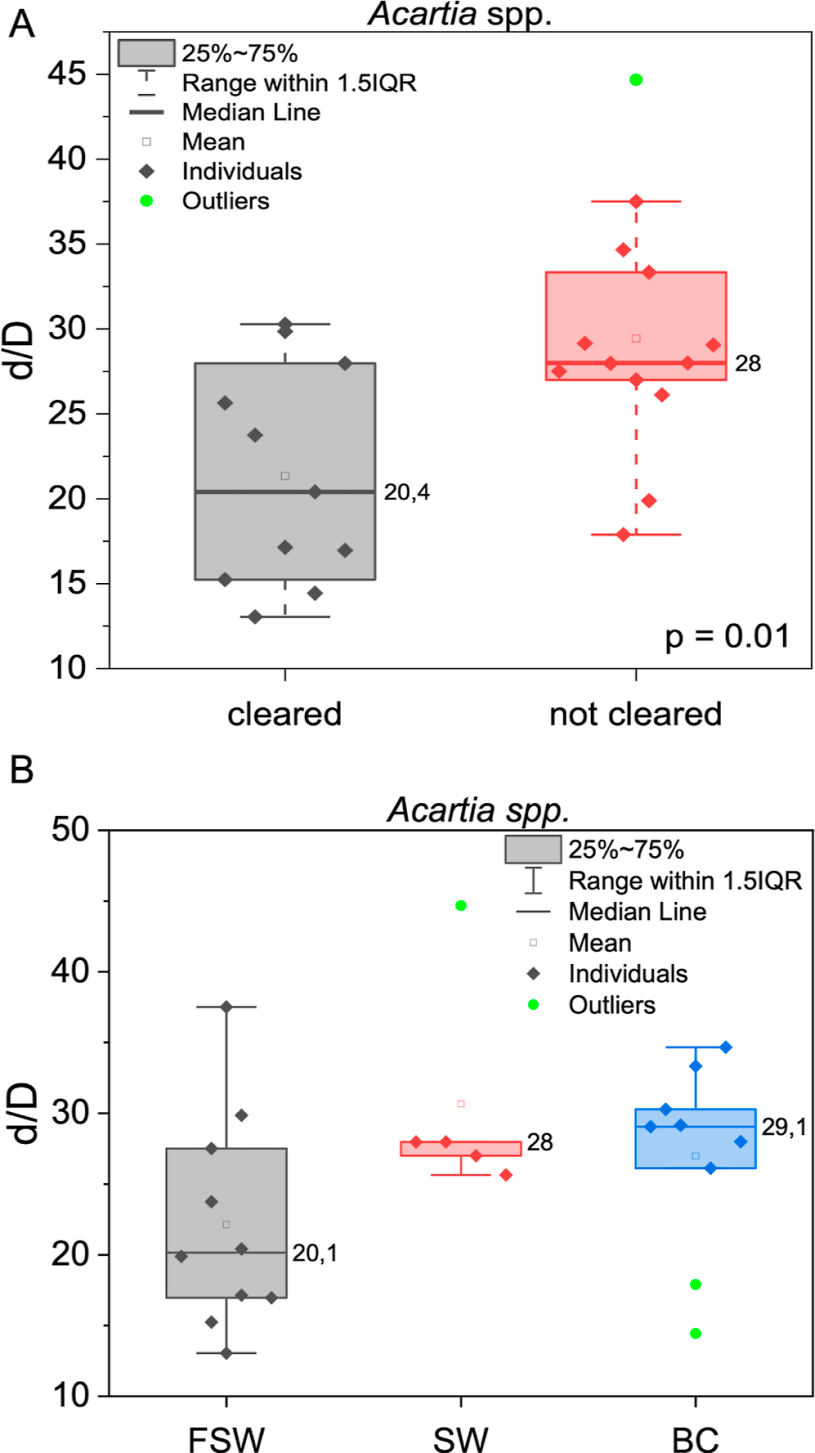
Box plots of the intestinal-to-body transverse ratio (d/D) measured at the midgut B-cell region of *Acartia spp.* copepods. (A) Comparison between cleared guts (*n* = 11) (no detectable pigments or BC emission) and noncleared guts (*n* = 13) (presence of food-associated yellow emission at 560 nm or broad DBC-like fluorescence). Median d/D values indicate progressive intestinal swelling from cleared to noncleared individuals (*p* < 0.05). (B) Distribution of d/D ratios under three experimental conditions: filtered seawater (FSW, cleared control) (*n* = 10), natural seawater (SW, food-rich control) (*n* = 5), and BC-exposed individuals (BC and PBC) after 24 h of gut clearing in FSW (*n* = 8). Median d/D values increase from cleared to feeding/BC-exposed groups, consistent with active ingestion and gut distension. Boxes show interquartile ranges (IQRs), whiskers extend to 1.5 × IQR, and green circles denote outliers. Sample sizes: FSW (*n* = 10), SW (*n* = 5), BC + PBC (*n* = 9). No significant difference was found between food-fed and BC-fed copepods (Kruskal–Wallis, *p* > 0.05).

Guts were classified according to their spectral fingerprints under 2P excitation. Cleared guts displayed only blue-green chitin-like emission near 495 nm (Figure 2C), confirming complete intestinal clearance. Noncleared guts showed either (i) narrow yellow emission near 577 nm or a red peak at ∼670 nm, typical of natural food pigments in the SW group, or (ii) broad DBC-like emission in BC-exposed copepods.

As shown in Figure 3A, the mean d/D ratio increased from 0.20 to 0.28 between cleared and noncleared guts, indicating a significant correlation (*p* < 0.05) between intestinal distension and retained material. This swelling reflects both gut filling and the activation of digestive B-cells, consistent with vacuolar expansion observed during feeding.^27^ In Figure 3B, mean d/D values were ∼0.20 for FSW, ∼0.28 for BC and PBC, and ∼0.29 for SW. The progressive increase from cleared to feeding and BC-exposed conditions confirms that soot ingestion elicits physiological responses comparable to natural feeding.

Complementary data in Table S1 show that copepods maintained in FSW exhibited a 70% ± 15% intestinal clearance after 24 h, whereas SW-fed individuals reached 40% ± 22%. BC-exposed copepods displayed a 33% ± 17% clearance but 67% ± 16% BC uptake, indicating active ingestion under food-deprived conditions.^52,53^ The absence of statistically significant differences between the groups in Figure 3B likely reflects the limited sample size and the presence of cleared-gut outliers (∼33%) within the BC-exposed sets. Nevertheless, the consistent d/D trend links intestinal swelling to both food availability and BC ingestion, with interindividual variability attributed to natural feeding rhythms and experimental stress.

Figure S4 compares copepods exposed to unprocessed BC (PBC + DBC) and centrifuged PBC (PBC-enriched, DBC-depleted). No systematic differences in d/D were observed, indicating that soot ingestion occurs independently of the dissolved fraction. This supports the interpretation that DBC represents a minor component of diesel soot, whereas PBC aggregates (∼1 μm, bacterial-size range) are the primary ingested form. Both *A. clausi* and *A. tonsa* exhibited similar intestinal responses, supporting the species-independent behavior of the midgut B-cell region in *Acartia* zooplankton as a morphologically constrained compartment with conserved swelling amplitude across species (Figure S5).

Quantitative analysis of the d/D ratio in the midgut B-cell region thus reveals consistent physiological responses to both natural feeding and BC ingestion. Copepods in filtered seawater (FSW) showed the lowest ratios, confirming intestinal clearance and establishing a pigment-free baseline. In contrast, individuals in SW or exposed to BC displayed pronounced swelling, reflecting active ingestion of food particles or soot aggregates. The comparable intestinal expansion between BC and PBC groups demonstrates that copepods ingest particulate BC directly, not merely filtering dissolved or colloidal fractions. This supports the conclusion that DBC constitutes only a minor fraction of diesel soot, whereas particulate PBC, composed of ∼3 μ m soot aggregates, represents the dominant assimilated form.

The DBC-like emissions observed in posterior gut regions are therefore best interpreted as *in vivo* aggregation-dependent structural reorganization at the particle level of ingested PBC aggregates rather than uptake of pre-existing dissolved fractions. Such reorganization may involve partial disaggregation or colloidal dispersion facilitated by intestinal pH and enzymatic activity, which modulate intermolecular coupling and light–matter interactions. This interpretation aligns with theoretical and experimental findings that the aggregation state and morphology critically influence the optical absorption and refractive index of soot^54^ and that morphological compaction during soot aging alters absorption cross sections and emission behavior.^55^

Thus, 2P microscopy emerges not only as a high-resolution imaging tool but also as a selective photophysical probe capable of quantifying aggregation-dependent intestinal responses *in vivo*. Having established these quantitative indicators of BC ingestion and gut reorganization, the next section addresses the aggregation-dependent structural reorganization that drives the PBC-to-DBC-like spectral transition.

#### 3.2.4 Evidence for Aggregation-Dependent Spectral Changes of BC in Copepods

Building on the reference fingerprints established for PBC and DBC fractions (Figure 1), we analyzed the fate of ingested BC along the digestive tract of live *Acartia spp.* using spectral 2P imaging. Confocal z-stacks (side view, Figure 4A–E) revealed discrete BC-containing regions of interest (ROIs 1–5), progressing from the anterior foregut toward the posterior midgut and hindgut. Each ROI exhibited a characteristic emission profile that could be directly compared to the PBC reference spectrum (red-shifted maximum near ∼650 nm) and the broad green emission of DBC (∼550 nm).

**Figure 4:**
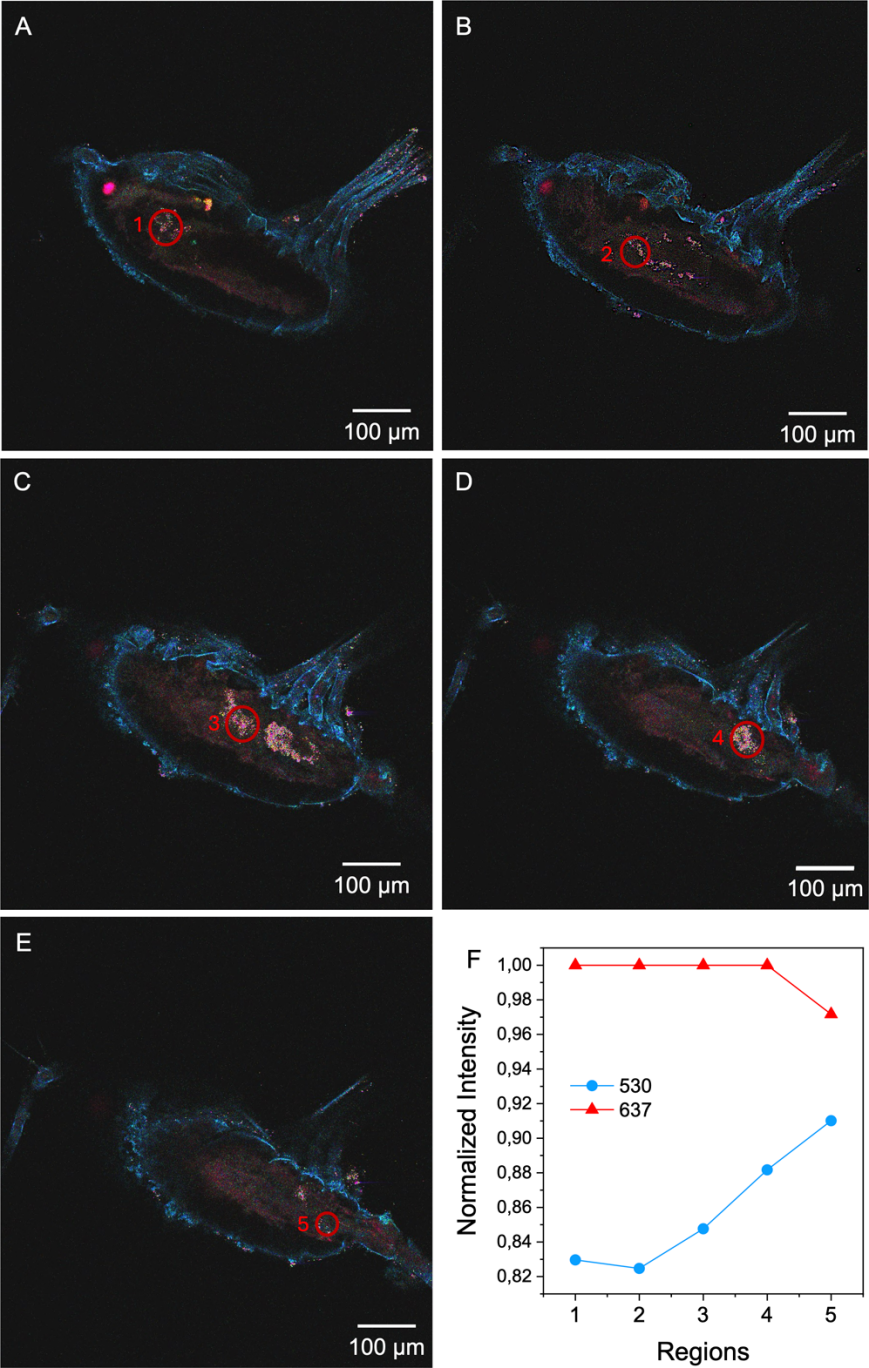
Aggregation-dependent spectral changes of BC along the copepod gut. (A–E) Sequential confocal sections (side view) of a live *Acartia spp.* exposed for 24 h to particulate BC (≈2.5 mg·L^–1^) following gut clearing. Each plane resolves a distinct gut segment, from anterior (A) to posterior regions (E). Emission progressively shifts from red PBC-like spectra (∼650–675 nm) in the anterior gut to green-centered DBC-like fluorescence (∼530–550 nm) toward the posterior tract. (F) Normalized emission intensities extracted from the ROIs in panels A–E show a continuous decrease of the PBC band (637 nm red curve) and a gradual increase in the DBC band (530 nm blue curve), evidencing *in vivo* reorganization of ingested BC aggregate rates.

To quantify these variations, spectra were normalized to the emission maximum, and intensities were extracted at 530 nm (DBC band) and 637 nm (PBC band). Plotting these values as a function of gut position (Figure 3F) showed a clear progression: BC entering the anterior gut displayed predominantly PBC-like emission; further along the digestive tract, however, the DBC-associated band increased gradually, reaching ∼10% of the ROI 1 value, while the relative PBC contribution decreased.

This systematic redistribution indicates that soot-like PBC aggregates undergo an aggregation-dependent structural reorganization under intestinal conditions, which modifies their photophysical emission signatures. Because diesel exhaust material is initially dominated by particulate soot with only minor dissolved fractions, the increase in DBC-like signatures within copepods likely reflects an *in vivo* transformation, not the uptake of pre-existing DBC. These results are consistent with a scenario in which the intestinal microenvironment partially disrupts or relaxes strongly coupled aromatic domains, reducing the density of low-energy states and shifting the emission toward the broader, higher-energy DBC-like profile.

Although digestive enzymes in *Acartia spp.* play a key role in feeding cycles, previous histochemical studies^27^ indicate that midgut B-cells primarily regulate secretion and vacuolar dynamics rather than performing enzymatic breakdown of refractory particles. Thus, the observed PBC-to-DBC-like transitions are unlikely to arise from biochemical degradation. Instead, they point to a physico-photophysical reorganization driven by intestinal conditions—mild acidity, biosurfactants, weak oxidative activity, or the presence of amphiphilic compounds that can modulate intermolecular π–π coupling within soot aggregates. These conditions may not break PAH structures but can loosen stacked domains, increasing disorder and yielding the DBC-like emission observed *in vivo*. The mechanism is therefore best interpreted as a reversible aggregate-level reorganization, not a chemical decomposition of BC.

Depth-resolved spectral evolution could be fully documented in a limited number of specimens because of the intrinsic constraints of *in vivo* spectral imaging of motile zooplankton. Nevertheless, qualitative inspection across multiple individuals revealed the consistent occurrence of DBC-like emission signatures within the digestive tract. The longitudinal profile shown in Figure 4 should therefore be interpreted as a representative case illustrating the tendency toward spectral redistribution during gut transit rather than as a statistically averaged gradient.

#### 3.2.5 Ruling Out Spectral Filtering and Depth-Dependent Artifacts

Because the copepod was slightly tilted in the spherical observation chamber, the z-stack in Figure 4A–E captured consecutive anatomical compartments—from the anterior foregut to the posterior hindgut. Light emitted from each segment passed through thin layers of tissue before reaching the detector, raising a potential concern about wavelength-dependent attenuation (spectral filtering). In principle, the Rayleigh/Mie scattering (∝ λ^–*n*^) and pigment absorption (stronger in the UV–blue) would preferentially reduce short-wavelength emission with increasing depth, artificially enhancing red components.

However, the experimental results show the opposite: the green DBC band (530–550 nm) increases toward the posterior gut, while the red PBC band decreases (Figure 4F). This inverse trend is incompatible with a depth-related filtering effect during image acquisition and therefore confirms a genuine change in the BC photophysical state.

Additional controls further eliminate optical artifacts. The cleared-gut specimen in Figure 2C–C′, imaged under identical settings, displayed uniform chitin-like emission (∼495 nm) throughout the intestinal tract, without evidence of wavelength-dependent enhancement or suppression. Although this is a single optical plane rather than a z-stack, it effectively serves as a negative control, demonstrating that neither tissue curvature nor optical path length induces a systematic spectral bias.

Moreover, all spectra were collected with identical excitation power, pixel dwell time, and detector gain, and normalized either to the red shoulder (∼637 nm). Cross-system calibration of the GaAsP detector arrays (Methodology section) ensured uniform spectral sensitivity. Together, these controls confirm that the enhancement of the DBC band in the posterior gut reflects true *in vivo* reorganization at the particle-aggregate level, rather than an artifact of light scattering or filtering.

Our results demonstrate that diesel-derived PBC undergoes redistribution along the copepod digestive tract, accompanied by aggregation-dependent structural reorganization under intestinal physicochemical conditions. Such processes, including partial disaggregation and enhanced colloidal dispersion, are consistent with previous evidence that the stability of soot-derived suspensions is strongly modulated by environmental factors, such as pH and surfactant-like organic matter.^56,57^ These transformations may increase the relative abundance of dissolved or colloidal BC (DBC), a fraction characterized by greater surface reactivity and potential bioavailability.

Recent global assessments indicate that oceanic stocks of PBC are substantially lower than expected from atmospheric and riverine inputs, suggesting the existence of underestimated removal pathways.^58^ Our findings support the hypothesis that biological processing and physicochemical restructuring in marine food webs can contribute to the redistribution and apparent loss of particulate soot in the ocean. By revealing how the aggregation state governs the photophysical detectability and environmental behavior of combustion-derived carbon, this study provides new mechanistic insight into the marine cycling of BC and highlights the value of nonlinear spectral microscopy for probing pollutant transformations *in vivo*.

## Supplementary Material



## Notes

2P spectral images and DLS size distributions of dissolved (DBC) and particulate (PBC) BC fractions (Figure S1); 2P spectral image of *A. tonsa* after 24 h exposure to unfiltered seawater, with corresponding emission spectra from defined gut ROIs (anterior, midgut zone II, posterior gut, and B-cells) (Figure S2); definition and illustration of the standardized morphometric metric (gut-to-body diameter ratio, *d/D*) measured at the midgut B-cell region in *Acartia spp.* (Figure S3); box-plot analysis of intestinal swelling (d/D) in BC versus PBC-exposed copepods, showing comparable responses across particulate fractions (Figure S4); and species-level comparison of intestinal swelling (d/D) in *A. clausi* and *A. tonsa* across cleared and noncleared gut states, demonstrating conserved swelling amplitudes independent of species or clearance condition (Figure S5) (PDF)
