# Supplementary material for: Label-Free Two-Photon Spectral Microscopy to Track Black Carbon Fate in Live Copepod
Source: Environ Sci Technol. 2026 May 25;60(22):16135–45. doi: 10.1021/acs.est.5c18449 (PMC13262048; doi:10.1021/acs.est.5c18449)
Supplement: Supplementary file 1 [file es5c18449_si_001.pdf]

Supporting Information
Label-free two-photon spectral microscopy to track Black Carbon fate in live copepod

Maria L. F. Vicente*[1,2], Jeanne Blanchet[2], Mariana M. Veras[3], Dominique Jamet[2], Jean-Louis Jamet[2], Stéphane Mounier[2], Houssam Hajjoul*[2], and Francisco E. G. Guimarães*[1]

[1]São Carlos Institute of Physics, University of São Paulo, São Carlos, 13566-590, Brazil
[2]Univ Toulon, Aix Marseille Univ, CNRS, IRD, MIO, Toulon, 83041, France
[3]Laboratório de Patologia Ambiental e Experimental, LIM05 – Hospital das Clinicas, Faculdade de Medicina da Universidade de São Paulo, São Paulo, 01246-903, Brasil

*Email: maria.luiza.vicente@usp.br;hajjoul.houssam@univ-tln.fr;guimaraes@ifsc.usp.br

Summary

9 Pages
1 Supporting Text
1 Supporting Tables
5 Supporting Images

Text S1

Two-photon Absorption of Isolated Black Carbon Nanoparticles

To quantify the optical interaction of soot nanoparticles with the laser field, transmission measurements were performed on isolated primary soot nanoparticles (∼30 nm diameter) because these units constitute the fundamental optically interacting substructures of larger BC aggregates. These measurements were carried out using 2P excitation in the range 690 nm – 1100 nm of a tunable laser. In hierarchical soot agglomerates, nonlinear optical excitation is governed primarily by local absorption within these primary domains rather than by the overall aggregate size. The transmitted intensity $T(\lambda) = I/I_0$ was recorded from 700–1100 nm under constant laser fluence. From the absorbance $A(\lambda) = \ln(1/T(\lambda))$, the contributions of one-photon scattering and two-photon absorption were separated. The scattering term $S(\lambda)$ was obtained by fitting a power law $S(\lambda) \propto \lambda^{-n}$ to the near-infrared tail (700–1100 nm), yielding n≈1, consistent with Mie/Rayleigh behavior for small carbonaceous particles. The residual component was attributed to two-photon absorption, allowing estimation of the two-photon absorption coefficient $\beta(\lambda)$. The 800 nm transmission-mode measurements on isolated nanoparticles indicated that only a minor fraction of the incident energy contributes to two-photon absorption ∼3%. Further details regarding these findings are available in Maria Luiza Ferreira Vicente, Sebastião Pratavieira, Mariana Matera Veras, Paulo Saldiva, Houssam Hajjoul, Francisco Eduardo Gontijo Guimarães, "Two-photon excitation as a selective tool to characterize black carbon nanoparticles in the environment," Proc. SPIE 12999, Optical Sensing and Detection VIII, 1299918 (20 June 2024); https://doi.org/10.1117/12.3022330.

Tables

Table S1: Mean BC/PBC uptake and intestinal clearance across experimental groups (FSW, SW, BC, PBC). Filtered controls (FSW) exhibited the highest clearance levels (70 ± 15%), confirming effective gut emptying and providing a reliable baseline for comparison with BC-exposed individuals. In contrast, copepods exposed to BC or PBC showed reduced clearance (33 ± 16%) together with substantial particle uptake (67 ± 16%), demonstrating active ingestion even under food-deprived conditions. These uptake levels are sufficient to resolve the aggregation-dependent spectral reorganization of BC within the gut and to support the interpretation of *in vivo* biotransformation processes.

**Group**	**n**	**% of BC uptake**	**SE (%)**		**% of clearance SE (%)**
FSW	10	-	-	70	15
SW	5	-	-	40	22
BC	9	67	17	33	16

Figures

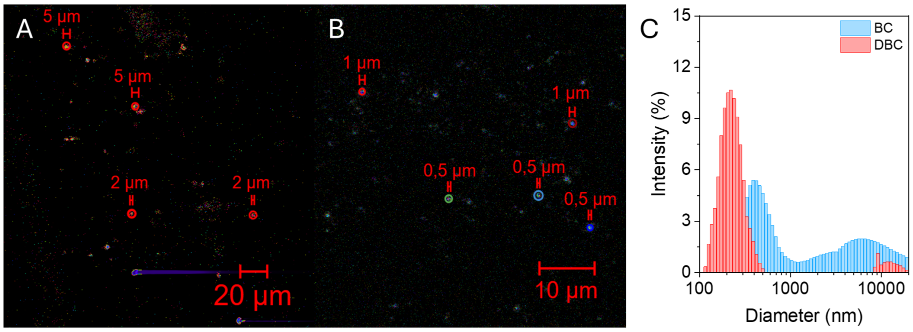

Figure S1: Morphological and size characterization of diesel-derived Black Carbon fractions. (A–B) Two-photon confocal images (spectral mode) of BC deposited on glass after suspension in filtered seawater. Red circles mark particulate BC (PBC; ∼1–5 μm) and blue/green circles mark submicron domains consistent with dissolved/colloidal BC (DBC; ∼0.5 μm). Scale bars: 20 μm (A) and 10 μm (B). (C) Dynamic Light Scattering (DLS) intensity distributions for the unfiltered BC suspension (blue) and the 0.45 μm filtrate representing the DBC fraction (red). The BC suspension exhibits a broad multimodal distribution spanning ∼300 nm to 10 μm, whereas the DBC filtrate shows a dominant submicron population (∼150–400 nm). Intensity values reflect scattering amplitude and are not proportional to particle number.

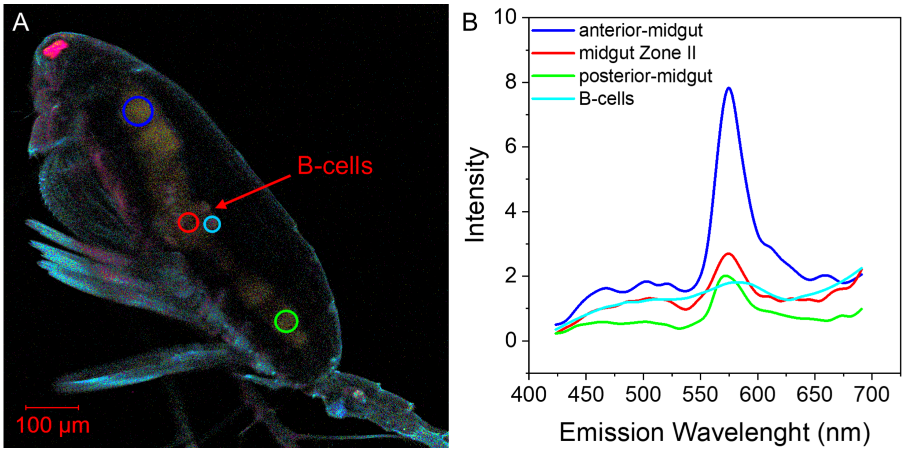

Figure S2: Spectral characterization of food-filled gut content in *Acartia tonsa*. (A) Two-photon spectral image (lateral view) of a copepod incubated for 24 h in unfiltered seawater (SW), showing a filled intestinal tract with characteristic yellow-orange emission. (B) Emission spectra extracted from the indicated regions of interest (ROIs), anterior midgut, midgut Zone II, posterior midgut, and B-cells, revealing a consistent, intense, narrow peak at ∼577–580 nm assigned to astaxanthin. Broader red contributions extending toward ∼680–700 nm correspond to chlorophyll *a*. The simultaneous presence of both pigments reflects ingestion of cyanobacteria and other natural dietary components.

The B-cells (red arrow, cyan ROI in panel A) display a broadened spectrum lacking sharp monomeric peaks, consistent with digestive vacuolar activity and pigment aggregation during active feeding. These results confirm robust feeding behavior and validate the SW group as a positive control for food-induced gut swelling.

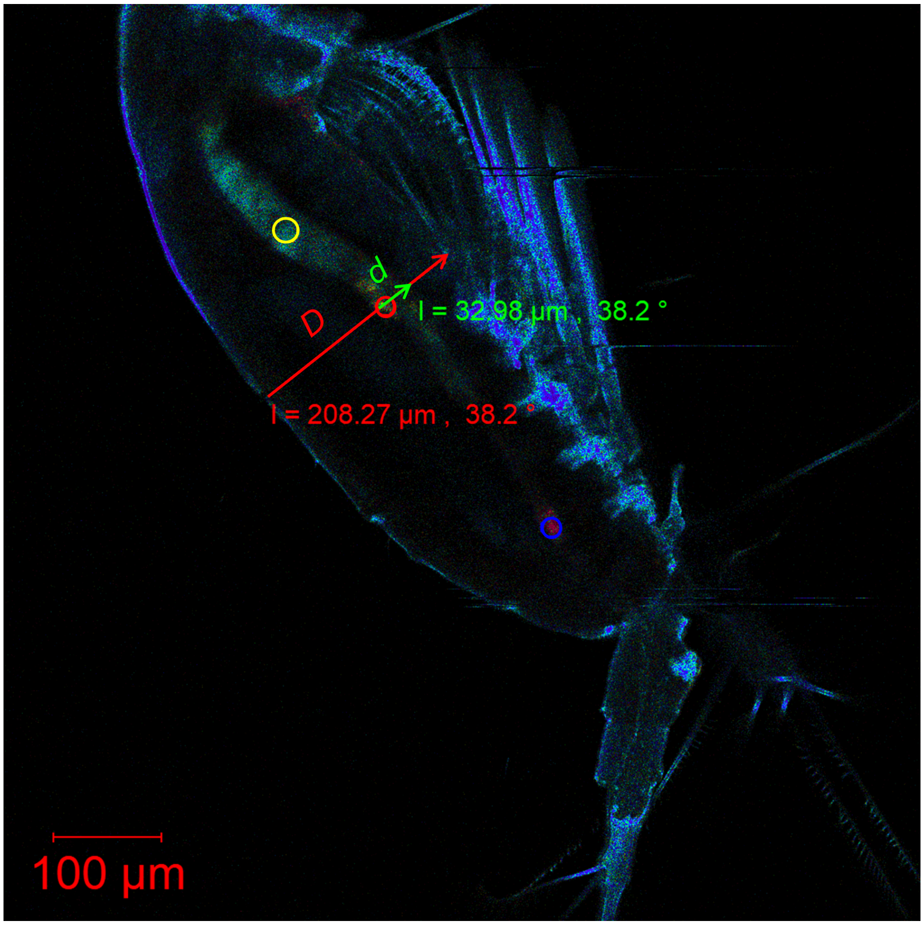

Figure S3: Morphometric quantification of intestinal swelling in *Acartia* spp. Illustration of the measurement procedure used to quantify gut distension. The transverse intestinal diameter (d) and the corresponding body diameter (D) were extracted from the same anatomical plane in the midgut B-cell region. This region was selected because it contains (i) the characteristic fluorescence of B-cells and (ii) a consistent positional landmark near the first pair of swimming legs. Using these two features ensured a standardized and reproducible measurement across individuals of different sizes. The gut-to-body ratio (d/D) was then used as a dimensionless metric of gut swelling for all experimental groups.

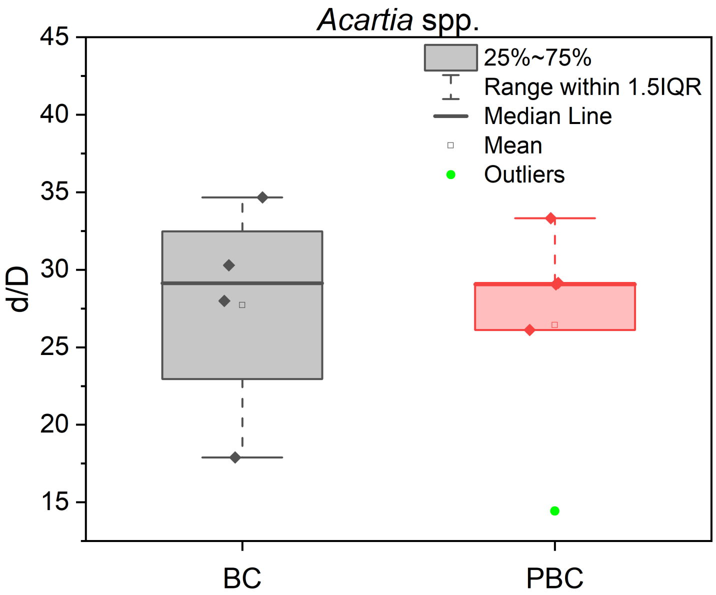

Figure S4: Intestinal swelling in copepods exposed to BC and PBC. Box plots of the gut-to-body transverse ratio (d/D) measured in the midgut B-cell region for copepods exposed to unfiltered BC (PBC + DBC) and to the particulate-enriched fraction (PBC). Boxes represent the interquartile range (IQR), horizontal lines indicate medians, whiskers extend to 1.5 x IQR, and green points denote outliers. No significant difference was detected between groups (Kruskal–Wallis, $p > 0.05$; $n = 9$), with both conditions showing comparable median d/D values ($\sim$0.28). These results indicate similar intestinal swelling responses regardless of the presence or absence of the dissolved/colloidal DBC fraction.

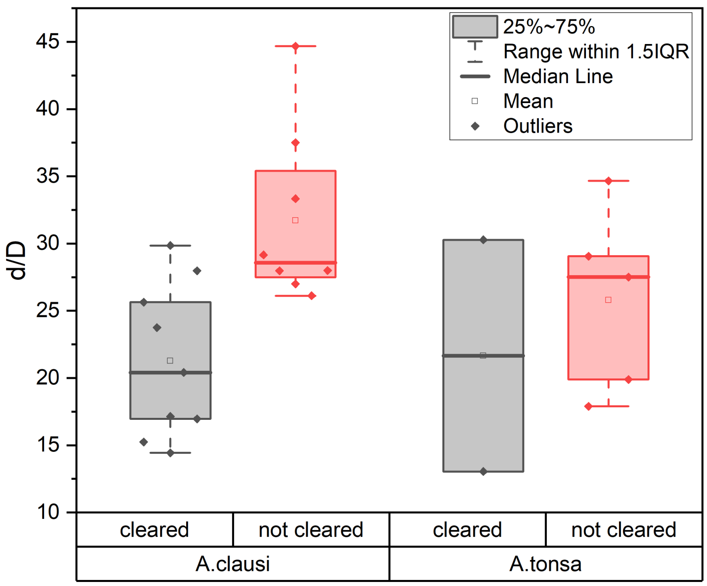

Figure S5: Intestinal swelling across clearance stages in *Acartia clausi* and *Acartia tonsa*. Box plots of the gut-to-body transverse ratio (d/D) measured in the midgut B-cell region for both species under cleared and not-cleared conditions (pooled across FSW, SW, BC, and PBC groups). Boxes represent the interquartile range (IQR), horizontal lines indicate medians, whiskers extend to 1.5× IQR, and symbols denote means and outliers.

Both species showed comparable d/D values for not-cleared guts (∼0.27–0.29) and cleared guts (∼0.20–0.21), indicating similar feeding responses and intestinal swelling amplitudes. No statistically significant differences were detected between *A. clausi* and *A. tonsa* (Kruskal–Wallis, $p > 0.05$), suggesting that the midgut B-cell region behaves as a morphologically conserved compartment across these calanoid copepods.
